# Folate-functionalized SMMC-7721 liver cancer cell membrane-cloaked paclitaxel nanocrystals for targeted chemotherapy of hepatoma

**DOI:** 10.1080/10717544.2021.2015481

**Published:** 2021-12-28

**Authors:** Wenwen Shen, Shuke Ge, Xiaoyao Liu, Qi Yu, Xue Jiang, Qian Wu, YuChen Tian, Yu Gao, Ying Liu, Chao Wu

**Affiliations:** a Pharmacy School, Jinzhou Medical University, Jinzhou, China; b Department of Emergency Management, Liaoning Provincial Center for Disease Control and Prevention, Shenyang, China; c Department of Medical Oncology, The Third Affiliated Hospital of Jinzhou Medical University, Jinzhou, China; d Department of Medical Oncology, The First Affiliated Hospital of Jinzhou Medical University, Jinzhou, China

**Keywords:** Paclitaxel, nanocrystal, folate functionalized, SMMC-7721 liver cancer cell membrane, targeted therapy

## Abstract

In this study, we prepared a folic acid-functionalized SMMC-7721 liver cancer cell membrane (CM)-encapsulated paclitaxel nanocrystals system (FCPN) for hepatoma treatment. Transmission electron microscopy (TEM) characterization showed that FCPN was irregular spherical shapes with a particle size larger than 200 nm and a coated thickness of approximately 20 nm. In an *in vitro* release experiment, FCPN indicated a slowly release effect of paclitaxel (PTX). Cell experiments demonstrated that FCPN was taken up by SMMC-7721 cells and significantly inhibited the proliferation of SMMC-7721 cells, which illustrated that FCPN had good targeting ability compared with PN and CPN. According to the results of *in vivo* animal experiments, FCPN significantly inhibited tumor growth. Tissue distribution experiments proved that FCPN could accumulate significantly in tumor tissues, which further explained why FCPN had good targeting ability. These results clearly suggested that folate-functionalized homotypic CM bionic nanosystems might represent a very valuable method for liver cancer treatment in the future.

## 1. Introduction

Liver cancer is a common malignant tumor worldwide, with a 5-year survival rate of less than 10% (Cheng et al., 2018). Chemotherapy is the most common strategy for liver cancer (Wang et al., 2018). As a commonly used chemotherapy drug, paclitaxel (PTX) is effective for the treatment of liver cancer and can inhibit tumor cell mitosis by stabilizing tubulin (Raza et al., 2019). Moreover, it can effectively treat lung cancer, gastric cancer, ovarian cancer, breast cancer and glioma (Wu et al., 2018). However, it is almost insoluble in water, which affects its bioavailability and causes difficulty in clinical application. Therefore, the improvement of solubility and targeting ability is very important to enhance its therapeutic effect.

The rapid development of nanotechnology provides new ideas for the treatment and diagnosis of cancer (Manzur et al., 2017; He et al., 2018; Dong et al., 2019). Currently, various nanocarriers, such as mesopore silica (He et al., 2017), liposomes (Eloy et al., 2020), carbon nanotubes (Soleyman et al., 2015), polymer nanoparticles (Levit et al., 2020), and cyclodextrin nanoparticles (Yan et al., 2019), have been widely incorporated with PTX to improve its solubility and targeting ability. However, these methods generally have problems including low drug loading and targeting ability. In recent years, PTX nanocrystals (PNs) have attracted extensive research interest due to their high drug content and good water solubility (Ni et al., 2015; Lu et al., 2016; Huang et al., 2021). The principle is that nanocrystals have nanoscale dimensions. According to the Ostwald–Freundlich and Noyes–Whitney equations, the smaller the particle size is, the larger the specific surface area (Lu et al., 2014; Wu et al., 2018). This is closely related to the water solubility of the drug. Moreover, due to passive targeting of nanocrystals, more drug is delivered and accumulates in tumor tissue. However, this is not sufficient. The low specificity of passive targeting makes it impossible to distinguish normal cells and tumor cells, which leads to severe adverse reactions.

To solve this problem, PNs with active targeting functions have been thoroughly researched. Herceptin was used to modify docetaxel nanocrystals to enhance the therapeutic effect of docetaxel on HER2-positive breast cancer (Noh et al., 2016). Folic acid (FA) and polyethylene glycol modified PNs to improve the targeting treatment effect of PTX on breast cancer (Zhao et al., 2021). RGD and polyethylene glycol functioned as PNs to enhance the targeting ability of PTX for A549 lung adenocarcinoma (Huang et al., 2019). Recently, cell membranes (CMs) have emerged as new delivery materials for antitumor drugs (Li et al., 2018; Zou et al., 2019; He et al., 2020). This kind of biomimetic nanoparticle can facilitate a long cycle and is conducive to the absorption of drugs (Hu et al., 2020; Liu et al., 2020; Mao et al., 2021); for instance, platelet membrane-coated PNs enhance the effect of postoperative chemotherapy (Mei et al., 2020). As the technology has developed, studies found that some CMs have good active targeting. For instance, white CM biomimetic nanoparticles can target tumor inflammatory sites (Parodi et al., 2013), platelet membrane biomimetic nanoparticles can target damaged blood vessels after surgery (Mei et al., 2020), and cancer CM biomimetic nanoparticles can target homologous tumor sites (Harris et al., 2019).

In this study, inspired by homologous targeting of cancer CMs, we designed a PTX nanocrystalline particle coated with SMMC-7721 liver cancer CM for liver cancer targeting treatment. FA is one of the most common small molecules targeting ligands that has been widely investigated and has a good affinity for folate receptors (FRs), which are overexpressed in the liver cancer cells (Cao et al., 2020; Gao et al., 2020). To further strengthen the targeting of the drug delivery system, FA was modified on the surface of the SMMC-7721 CM to obtain folate-functionalized SMMC-7721 liver cancer CM-cloaked PTX nanocrystals (FCPN). FCPN has the characteristics of good active targeting effects, avoidance of immunocapture, long circulation, high bioavailability, and few side effects. Through *in vitro* cell experiments and *in vivo* animal experiments, we examined the antitumor potential of FCPN to verify the active targeting capability of the folate-functionalized cancer CM. This work is highly meaningful for cancer treatment.

## 2. Materials and methods

### 2.1. Materials

Paclitaxel was ordered from Xi’an Natural Field Bio-Technique Co, Ltd. (Xi’an, China) with >99% purity. Folic acid, N-hydroxysuccinimide (NHS), dicyclohexyl carbodiimide (DCC), dimethyl sulfoxide (DMSO), sodium dodecyl sulfate (SDS), ethanol, methanol, paraformaldehyde, and Pluronic 127 (F127) were purchased from Aladdin Chemical Reagent Co. (Shanghai, China). Thiazolyl blue tetrazolium bromide (MTT), propidium iodide (PI), Annexin V-FITC apoptosis detection kit, trypsin, Triton X-100, and bovine serum albumin were supplied by Nanjing Jiancheng Bioengineering Institute (Nanjing, China). SMMC-7721 cell lines were obtained from the National Experimental Cell Resource Platform (Beijing, China). RPMI 1640 medium and fetal bovine serum (FBS) were purchased from Beijing Dingguo Changsheng Biotech Co. Ltd. (Beijing, China). Phenylmethanesulfonyl fluoride (PMSF), 4′,6-diamidino-2-phenylindole (DAPI), mitochondrial membrane potential assay kit (JC-1), calcein-AM/PI were provided by Beyotime Biotechnology Co. (Shanghai, China). Bcl-2, Bax, and cleaved caspase-3 were obtained from Abcam (Cambridge, UK). N-Cadherin, galectin-3, EpCAM, CD147, and EGFR were purchased from Wanleibio (Shenyang, China).

### 2.2. Cell culture

The liver cancer cell line SMMC-7721 was cultured with a nutrient solution composed of 90% RPMI-1640 medium, 1% penicillin, 1% streptomycin, and 10% FBS. Cell digestion was performed using 0.25% trypsin during cell passage. FBS (90%) and DMSO (10%) were used for cryopreservation. The cells were finally stored at −80 °C.

### 2.3. Preparation of FCPN

#### 2.3.1. Extraction of CM

First, the liver cancer cell line SMMC-7721 was cultured in a 15 mL cell culture flask. When the cells were overgrown, after digestion and centrifugation, 15 mL of 0.2× PBS was added to the centrifuge tube to swell and burst the cells for 24 h. Then, after centrifugation at 1250 r/min for 10 min, the supernatant was discarded. A solution (3 mL) containing 1 mM NaHCO_3_ and 0.7 mM EDTA was added to the centrifuge tube. In addition, 30 µL of PMSF was replenished, and then the cell suspension was transferred to a homogenizer for homogenization in an ice bath. Finally, CM was obtained by gradient centrifugation at 4 °C and stored at −20 °C.

#### 2.3.2. Preparation of FA-CM

First, 500 mg of FA was dissolved in 10 mL of DMSO with 0.25 mL of triethylamine. At room temperature, 0.25 g of DCC and 0.26 g of NHS were added to the FA solution and stirred overnight. Then, the solution was centrifuged to obtain the supernatant. A mixed solution containing 30% acetone and 70% ether was added to the above solution to obtain a yellow precipitate. The precipitate was washed three times with ether and dried under a vacuum to obtain afaint yellow solid powder, which was an FA active ester (Cao et al., 2020). FA active ester was dissolved in water and mixed with the CM to obtain FA-CM.

#### 2.3.3. Synthesis of PN, CPN, and FCPN

PTX nanocrystals were prepared by a combination of antisolvent precipitation and high-speed shearing (Wei et al., 2015; Chai et al., 2019). First, 40 mg of PTX was dissolved in 1 mL of ethanol as the organic phase. At room temperature, 200 mg of Pluronic F-127 (F127) was dissolved in 10 mL of water as the water phase. One milliliter of organic phase was injected into the water phase under rapid stirring (1200 rpm) and sonicated for 5 min. Then, the above suspension was sheared at a high speed of 12,000 rpm for 2 min by a high shear emulsification machine (FLUKO Technology Development Co., Ltd., Shanghai, China) to obtain PN. The PN was dried under vacuum for 2 h to remove ethanol.

PN was mixed with CM and FA-CM in a certain proportion. The system was sonicated for 5 min with a 30-second break in an ice bath and then incubated for 2 h at 37 °C to obtain CPN and FCPN. To study the optimal membrane ratio, FA-CM each with a protein concentration of 2 mg/mL were mixed with PN containing 2 mg, 4 mg, 6 mg, and 8 mg of PTX (Chai et al., 2019). The samples were incubated with FBS, and the optical density of particles was determined with a microplate reader (VERSAmax, Molecular Devices, Sunnyvale, CA) by measuring the absorbance at 560 nm for 2 h. Finally, a 5% glucose solution was added to the suspension of CPN and FCPN as a freeze-dried protective agent. After freeze-drying, the samples were stored in a refrigerator at 4 °C.

### 2.4. Determination of drug loading

Different pharmaceutical preparations (5 mg) were dissolved in methanol and diluted, and the content of the drugs was determined by HPLC (mobile phase was acetonitrile–water (50:50, v/v) solution, flow rate 1.0 mL/min, wavelength 227 nm). The drug loading was calculated by the following formula:

$$\mathrm{LC}\;(\text{loading content})=\frac{\left(\text{weight of loading drug}\right)}{\left(\text{total weight of nanocomposites}\right)}\times 100\%$$

### 2.5. Characterization

#### 2.5.1. Morphological structure identification

A scanning electron microscope (SEM) (JEOL JSM-7001F, Tokyo, Japan) was used to characterize the morphology of the unsheared nanocrystals. Transmission electron microscopy (TEM) (JEM-1200EX; JEOL, Tokyo, Japan) was used to observe the morphological structures of PN, CPN, and FCPN.

#### 2.5.2. Particle size distribution and surface potential

The sizes and zeta potentials of PN, CPN, and FCPN were obtained using a laser dispersion particle size analyzer (Nano-ZS90, Malvern, Malvern, UK).

#### 2.5.3. Differential scanning calorimetry (DSC) and X-ray diffractometer (XRD)

The solid state of F127, pure PTX, PN, and FCPN was detected by DSC and XRD. DSC was performed using a differential scanning calorimeter (DSC-60, Shimadzu, Kyoto, Japan) from 70 °C to 300 °C under N_2_ flow of 150 mL/min at a rate of 10 °C/min. XRD was carried out by an X-ray refractometer with Cu-K radiation (Rigaku Geigerfex XRD, Tokyo, Japan, 30 kV and 30 mA Philips).

#### 2.5.4. Characterization of cell membranes

The targeting effects of CPN and FCPN were achieved by CM surface proteins. Sodium dodecyl sulfate-polyacrylamide gel electrophoresis (SDS-PAGE) was used to characterize whether the encapsulation was successful. Membrane proteins were extracted from CM, CPN, and FCPN with RIPA lysis buffer and further measured with BCA analysis. Then, the protein sample and marker were added, and electrophoresis was performed in an electrophoresis device. After staining with Coomassie Brilliant Blue, image development was performed under a microscope and analyzed by Quantity One 1-D Analysis Software (Bio-Rad, Hercules, CA). In addition, western blotting was used to analyze SMMC-7721 CM proteins, including CD147 (Zhu et al., 2015), EGFR (Psyrri et al., 2012; Zhao et al., 2017), EpCAM, N-cadherin, and Galectin-3 (Chen et al., 2016; Liu et al., 2019), to further verify the coating process.

### 2.6. *In vitro* release study

The drug release experiment was carried out in a shaker, and phosphate-buffered saline (PBS, 200 mL, pH 7.4) containing 0.05% SDS was used as the release medium at 37 °C. PTX, PN, and FCPN (equivalent to 2 mg of PTX) were added to 200 mL of dissolution medium. Within a specified period of time, 2 mL samples were removed and filtered with a 0.22 µm organic filter membrane. At the same time, 2 mL of fresh release medium was replenished. The released drug content was detected by HPLC.

### 2.7. *In vitro* cell research

#### 2.7.1. Cellular uptake

The preparation process of FITC-labeled (PN) was as follows. First, PTX was dissolved in 1 mL of ethanol to form an organic phase. At room temperature, the surfactants F127 and FITC were dissolved in 10 mL of water as the water phase. Then, 1 mL of the organic phase was injected into the water phase under rapid stirring (1200 rpm) and sonicated for 5 min to obtain FITC-labeled PN. FITC-labeled PN was dried for 2 h under vacuum to remove ethanol. FITC-labeled PN was mixed with CM and FA-CM, and then the systems were sonicated and incubated for 2 h at 37 °C to obtain FITC-labeled CPN and FITC-labeled FCPN.

Cell uptake was detected by a confocal laser scanning microscope (CLSM, BioTek Instruments, Winooski, VT). SMMC-7721 cells were inoculated in Petri dish. FITC-labeled PN, CPN, and FCPN were added and incubated for 1 h. Cells were fixed in 4% paraformaldehyde solution for 10 min. Hoechst 33342 and rhodamine phalloidin were successively used to treat the cells for staining. Next, the cells were immersed in 1 mL of PBS and observed by CLSM.

Cell uptake was also detected by flow cytometry. SMMC-7721 cells were seeded in six-well plates and cultured for 24 h. FITC-labeled PN, CPN, and FCPN were added to the cells for 1 h. The cells were quantitatively analyzed by flow cytometry (Agilent Biosciences Inc., Santa Clara, CA).

To verify the targeting specificity of FCPN, FITC-labeled FCPN was cocultured with the human liver cancer cell line SMMC-7721, the human breast cancer cell line SK-BR-3 and the mouse glioma cell line C6 for 1 h. The uptake of CMs by these three types of cells was observed by CLSM.

#### 2.7.2. Cytotoxicity analysis

The toxicity of PTX, PN, CPN, and FCPN was determined by the MTT test. SMMC-7721 cells were counted and seeded into a 96-well plate with 5000 cells per well for 48 h. Then, pharmaceutical preparations with different drug concentrations (500 ng/mL, 250 ng/mL, 100 ng/mL, 50 ng/mL, 10 ng/mL, and 5 ng/mL) were added into a 96-well plate and incubated for 48 h. Thiazolyl blue tetrazolium bromide (MTT) at 5 mg/mL was added and incubated for 4 h under dark conditions. DMSO (150 μL) was added to 96 wells. The plate was placed in a shaker and shaken for 10 min under dark conditions. The absorbance value (OD) of formazan was measured at 492 nm with a microplate reader (VERSAmax, Molecular Devices, Sunnyvale, CA). Cell viability was calculated using the following formula:

$$\text{Cell viability}=\frac{{\mathrm{OD}}_{t}}{{\mathrm{OD}}_{c}}\times 100\%$$

OD_t_ represents the absorbance of cells in the drug preparation treatment group and OD_c_ represents the absorbance of cells in the control group.

#### 2.7.3. Live and dead cell staining

SMMC-7721 cells were cultured in 12-well plates. After treatment with PTX and PTX preparations (equivalent to 10 ng/mL PTX) for 24 h, 500 μL of calcein-AM/PI detection working solution was added to each well and incubated at 37 °C in the dark for 30 min. Then, the cells were observed under a fluorescence microscope (Leica, Wetzlar, Germany) (calcein-AM shows green fluorescence, Ex/Em = 494/517 nm; PI shows red fluorescence).

#### 2.7.4. Flow cytometric detection of apoptosis

SMMC-7721 cells were seeded in a six-well plate and incubated for 24 h at 37 °C in a 5% CO_2_ incubator. PTX, PN, CPN, and FCPN (equivalent to 10 ng/mL PTX) were added to six-well plates. After culturing for 48 h, the cells were digested with trypsin. Then, the collected cells were placed in another centrifuge tube, and 500 μL of binding buffer was added for resuspension. Five microliters of Annexin V-FITC and PI were added and mixed gently in dark conditions. Finally, the cell samples were tested with a flow cytometer (Agilent Biosciences Inc., Santa Clara, CA).

#### 2.7.5. Measurement of mitochondrial membrane potential (MMP)

SMMC-7721 cells were cultured in 12-well plates. The cells were treated with PTX, PN, CPN, and FCPN (equivalent to 10 ng/mL PTX) for 24 h. JC-1 staining working solution was added and incubated at 37 °C for 30 min. The cells were immediately observed under a fluorescence microscope (Leica, Wetzlar, Germany).

#### 2.7.6. Immunofluorescence to detect apoptosis

The cells were cultured in 24-well plates for 24 h and then treated with different pharmaceutical products (equivalent to 10 ng/mL PTX) for 24 h. The cells were fixed with 4% paraformaldehyde solution, permeabilized with Triton X-100, and blocked with goat serum for 1–2 h in turn. Then, we added cleaved caspase-3 to the cells overnight at 4 °C. The cells were incubated for 2 h with the secondary antibody, and the nuclei were stained with DAPI. Fluorescence microscopy was used to observe cell immunofluorescence.

#### 2.7.7. Western blot

SMMC-7721 cells were cultured in a 15 mL cell culture flask. The cells were treated with different preparations (equivalent to 10 ng/mL PTX) for 48 h. The adherent cells were scraped off on ice. The collected cells were treated with 300 µL of lysis buffer. After centrifugation, the total protein concentration of the collected supernatant was determined by BCA analysis.

Then, the protein samples were prepared and added to the sample well for polyacrylamide gel electrophoresis. Next, the polyacrylamide gel was moved onto the nitrocellulose membrane and blocked with BSA for blocking solution. After incubation with specific antibodies (Bcl-2, Bax, and cleaved caspase-3) and the secondary antibody at 4 °C, the film was processed with ECL developer. Quantity One 1-D Analysis Software (Bio-Rad, Hercules, CA) was used to analyze the expression.

### 2.8. *In vivo* experiment

#### 2.8.1. Establishment of mouse tumor model

Balb/c nude mice (females, 18–20 g) were purchased from Liaoning Changsheng Biotechnology Co., Ltd. (Benxi, China) and fed in an SPF-free environment. The experiment was carried out under the Animal Management Regulations of Jinzhou Medical University (2020). SMMC-7721 cells (5 × 10^6^) were injected into the vicinity of the right front leg of nude mice. A week later, there were noticeable tumor masses, which indicated that the tumor model was successfully established.

#### 2.8.2. In vivo antitumor effect and safety

When the tumor volume reached 300 mm^3^, nude mice were randomly divided into five groups (*n* = 5). Normal saline, PTX, PN, CPN, and FCPN (equivalent to PTX 20 mg/kg) were injected by the tail vein. Nude mice were injected once every three days for a total of seven times. Before each dose, we measured the body weight and the longest and shortest diameters of the tumor. The volume of the tumors was obtained according to the following formula:

$$\text{Volume of tumor}=\frac{(\text{longest diameter})\times {\left(\text{shortest diameter}\right)}^{2}}{2}\times 100\%$$

The tumor inhibition rate was obtained according to the following formula:

$$\text{Tumor inhibition rate}=(1-\frac{W_{t}}{W_{c}})\times 100\%$$

where *W*_c_ is the weight of the tumor in the saline group and *W*_t_ is the average weight of the tumor in each drug treatment group.

The mice were sacrificed after the last dose, and we removed the tumor tissue and the main organs (heart, liver, spleen, lung, and kidney). After that, the tumors and the main organs were fixed with paraformaldehyde and embedded in paraffin. The samples were sliced and were stained using ki67 for immunohistochemistry study. Hematoxylin and eosin (H&E) also were used to stain the sections to assess necrosis of tumor cells and safety of FCPN *in vivo*. The sections were observed with a fluorescence microscope (Leica DMI 4000B, Wetzlar, Germany).

#### 2.8.3. Biodistribution in the body

When the tumor volume reached 300 mm^3^, the tumors were randomly divided into four groups (*n* = 9). PTX, PN, CPN, and FCPN (equivalent to PTX 20 mg/kg) were injected into mice by the tail vein. Then, the tissues (heart, liver, spleen, lung, kidney, and tumor) of three mice were collected at each time point (2 h, 10 h, and 24 h). The heart, liver, spleen, lung, kidney, and tumor tissues were washed with saline, weighed, and then homogenized with DMSO. Two hundred microliters of tissue samples were transferred to 1.5 mL microcentrifuge tubes, and 20 µL of butyl paraben was added to each tube as an internal standard. Each mixture was vortexed for 5 min with 1 mL of methyl tert-butyl ether. After centrifugation at 12,000 rpm for 10 min, the organic layer was transferred to another test tube and evaporated in vacuum. Thirty microliters of methanol were added to each tube to redissolve the samples. PTX content was determined by HPLC.

#### 2.8.4. In vivo imaging

To further verify the targeting ability of FCPN, FITC-labeled PN, CPN, and FCPN were injected into the tail veins of tumor-bearing nude mice. After 3 h, the mice were sacrificed by cervical dislocation. *In vivo* fluorescence signals of different tissues (heart, liver, spleen, lung, kidney, and tumor) were observed using an *in vivo* imaging system (IVIS Spectrum, PerkinElmer, Waltham, MA) at excitation wavelengths of 518 nm and 494 nm.

#### 2.8.5. In vivo pharmacokinetic study

Male SD rats were purchased (180–220 g) from the Department of Laboratory Animal Science of Jinzhou Medical University for experiments. Twelve rats were randomly divided into three groups and fasted for 12 h. PTX, PN, and FCPN (equivalent to PTX 20 mg/kg) were injected into the rat body by tail vein. After administration, 1.5 mL of blood was taken from the fundus venous plexus at 0.083, 0.25, 0.5, 1, 2, 4, 8, 12, and 24 h. Whole blood was placed in a sodium heparin tube and centrifuged at 12,000 r/min for 5 min to separate the plasma. Butyl p-hydroxybenzoate was used as an internal standard, and methyl tert-butyl ether was used to extract PTX. The plasma drug concentration was determined by HPLC.

### 2.9. Statistical analysis

All data results were analyzed using GraphPad Prism (version 8.0, La Jolla, CA) and plotted using the mean ± SD. The data were analyzed to determine the difference in means between groups. *p*<.05 was considered statistically significant.

## 3. Results and discussion

### 3.1. The characterization of FCPN

**Figure 1.**
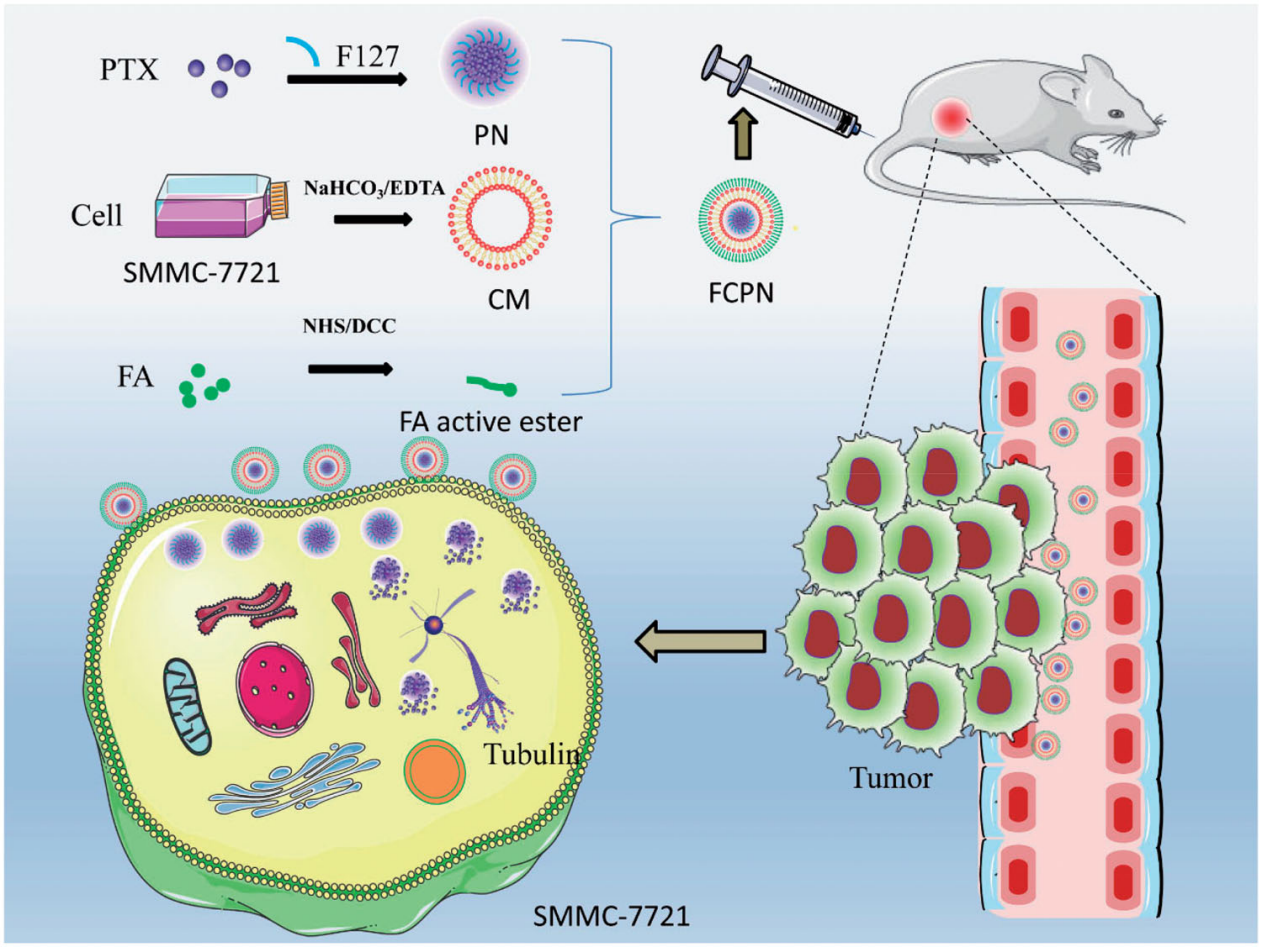
Schematic diagram of the FCPN preparation process and its antitumor effect.

**Figure 2.**
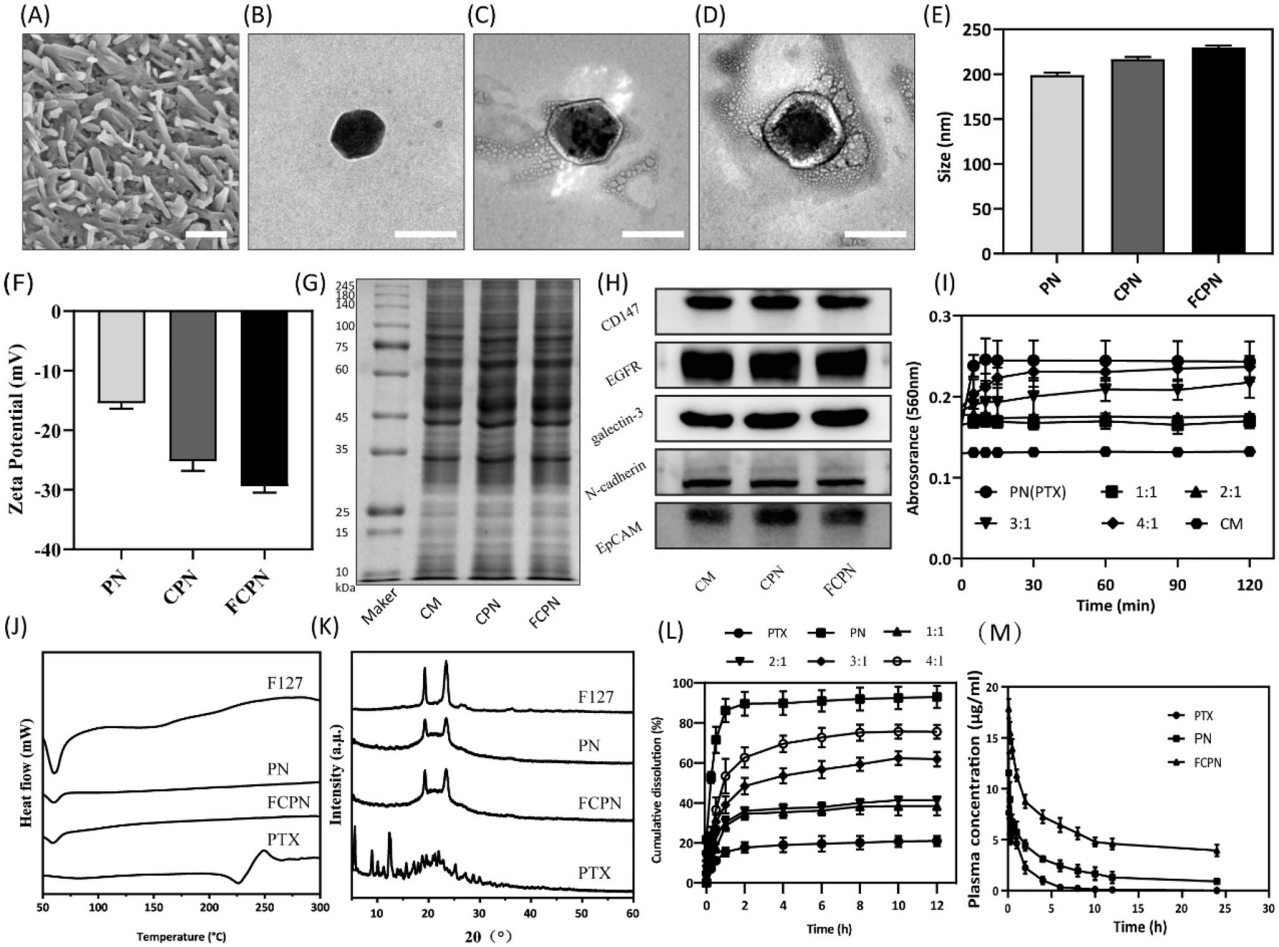
(A) SEM of PTX nanocrystals in non-sheared state. Scale bars: 1000 nm. (B–D) TEM images of PN, CPN, and FCPN. Scale bars: 200 nm. (E) The particle size distribution of PN, CPN, and FCPN by DLS. (F) The corresponding electric potential of PN, CPN, and FCPN. (G) SDS-PAGE analysis of CM, CPN, and FCPN. (H) Western blot analysis (e.g. EpCAM, N-cadherin, and galectin-3) of CM, CPN, and FCPN. (I) The study on membrane coating ratio (a ratio of PN containing PTX concentration and FA-CM containing protein concentration was 1:1, 2:1, 3:1, 4:1). (J) DSC curves of F127, PN, FCPN, and PTX. (K) XRD curves of F127, PN, FCPN, and PTX. (L) *In vitro* release of drugs in PBS (pH = 7.4) containing 0.05% SDS (a ratio of PN containing PTX concentration and FA-CM containing protein concentration was 1:1, 2:1, 3:1, 4:1). (M) The drug concentration–time curve *in vivo*. All data represented the mean ± SD (*n* = 3).

The synthetic schematic diagram of FCPN is drawn in Figure 1. PN was prepared with FI27 as the sole excipient by a combination of antisolvent precipitation and a high shear method. F127 is a novel type of polymer nonionic surfactant with good biosafety that can be used as a solubilizer and stabilizer during the preparation of nanocrystals (Liu et al., 2010; Lu et al., 2016). The cancer CM was extracted from SMMC-7721 cells and then connected with FA through an amide bond to obtain FA-CM. CM has a phospholipid bilayer structure, which is beneficial for improving the stability of drug molecules, like liposomes. In addition, CM has unique protein components and homotype aggregation targeting capabilities. Taking advantage of these advantages, it can enhance the targeting ability, systemic circulation time and stability of its coated particles (CPN). Different tumor cells have specific proteins that are highly expressed on the surface of the CM. This kind of protein can specifically recognize the corresponding tumor cells *in vivo*, allowing the PN coated in SMMC-7721 CMs to be more targeted to tumor tissue. It is well known that FRs are highly expressed in SMMC-7721 cells. Folic acid-functionalized CPN (FCPN) should further improve the active targeting of CPN and improve the antitumor effect of PTX. The prepared nanocrystals under a low shear state were characterized by SEM. The images showed a rod-like form with a diameter of approximately 200 nm and a length of approximately 1000 nm, as observed in Figure 2(A), which was consistent with the literature (Wei et al., 2015). TEM characterization indicated that the PN after high shear had an irregular spherical shape of approximately 200 nm, as shown in Figure 2(B). CPN and FCPN were irregular core–shell structures with a particle size larger than 200 nm, as shown in Figure 2(C,D). The particle size and zeta potential were obtained by DLS detection. The particle sizes of PN, CPN, and FCPN in Figure 2(E) were 199.17 ± 2.66 nm (PDI: 0.157), 218.83 ± 1.23 nm (PDI: 0.374), and 230 ± 2.10 nm (PDI: 0.356), respectively. The corresponding electric potentials in Figure 2(F) were −15.5 ± 0.89, −25.27 ± 1.53, and −29.37 ± 1.1, respectively. The changes in particle size and potential before and after coating and grafting proved the successful preparation of FCPN. The re-dispersion of CPN and FCPN after freeze-drying was investigated and the results in Figure S1 proved that the freeze-drying process did not affect the particle size and of CPN and FCPN. The seven days stability of CPN and FCPN in Figure S2 also indicated the particle size and electric potentials did not change significantly, which illustrated the preparation had good stability. The SDS-PAGE experiment in Figure 2(G) found that CPN and FCPN have electrophoretic bands consistent with those of SMMC-7721 liver cancer cell vesicles. Western blot further analyzed adhesion molecules (e.g. EpCAM, N-cadherin, and galectin-3) on tumor CMs and marker proteins (CD147, EGFR) of SMMC-7721 hepatocellular carcinoma cells. Adhesion molecules play an important role in improving the targeting effect. Through specific recognition of adhesion molecules, adhesion junctions are formed, which can make CPN and FCPN specifically bind with tumor cells. Figure 2(H) shows that biological adhesion factors (EpCAM, N-cadherin, and galectin-3) and membrane marker proteins (CD147 and EGFR) were highly enriched in CM, CPN, and FCPN. These results agree that the membrane coating process is successful and does not affect the activity of membrane surface proteins. The ratio of PN and the CM was optimized, and the results are provided in Figure 2(I). The best ratio achieved was 2:1. DSC and XRD were used to analyze the presence of drugs. The DSC curve in Figure 2(J) showed that the PN and FCPN characteristic endothermic peaks were visible at 59.8 °C, which was consistent with the characteristic endothermic peak of F127 and was obviously different from the crystal endothermic peak of raw PTX at 223 °C. In Figure 2(K), the XRD pattern indicated that raw PTX had obvious crystal diffraction peaks at 5.62°, 8.98°, 11.22°, and 12.36°. PN and FCPN only showed characteristic peaks at 19.3° and 23.5° and had no characteristic diffraction peak of raw PTX. The peaks at 19.3° and 23.5° were consistent with the characteristic diffraction peak of F127. These results illustrated that the PTX in PN and FCPN was amorphous, which directly contributed to the improvement of drug solubility. The drug loading of PN reached more than 93.8 ± 1.87% because there was almost no drug loss during the preparation of PN. For the freeze-dried samples of CPN and FCPN, the drug loading could also reach 39.33 ± 1.69% and 33 ± 1.41%, respectively. Compared with the high drug loading of PN, the decrease in drug loading for CPN and FCPN samples is due to the addition of lyophilized protective agent, but it was still higher than other preparations. The release experiment in Figure 2(L) demonstrated that PN had a rapid PTX release of 80% at 10 min in comparison with the 30% release of raw PTX at 12 h. Compared to other proportions of preparations, FCPN with a 2:1 ratio of PN and CM exhibited a slow-release effect and showed a lower PTX release of 40% at 12 h. This means that drugs have less loss after they enter the systemic circulation, which facilitates more drug delivery to the lesion site. The results of pharmacokinetic experiments in Figure 2(M) supported this view. The plasma half-lives (*t*_1/2_) of FCPN, PN, and PTX were 6.07 ± 0.85 h, 2.48 ± 0.31 h, and 1.27 ± 0.33 h, respectively. It is obvious that FCPN prolonged the action time of the drug, which effectively avoided being captured by the reticuloendothelial system. The AUCs_(0–∞)_ of FCPN, PN, and PTX were 166.23 ± 23.81, 34.64 ± 0.89, and 15.06 ± 2.31, respectively. The AUC_(0–∞)_ of FCPN was 11.04 times and 4.80 times that of free PTX and PN, respectively, which proved that FCPN effectively enhanced the bioavailability of PTX.

### 3.2. Targeting study

**Figure 3.**
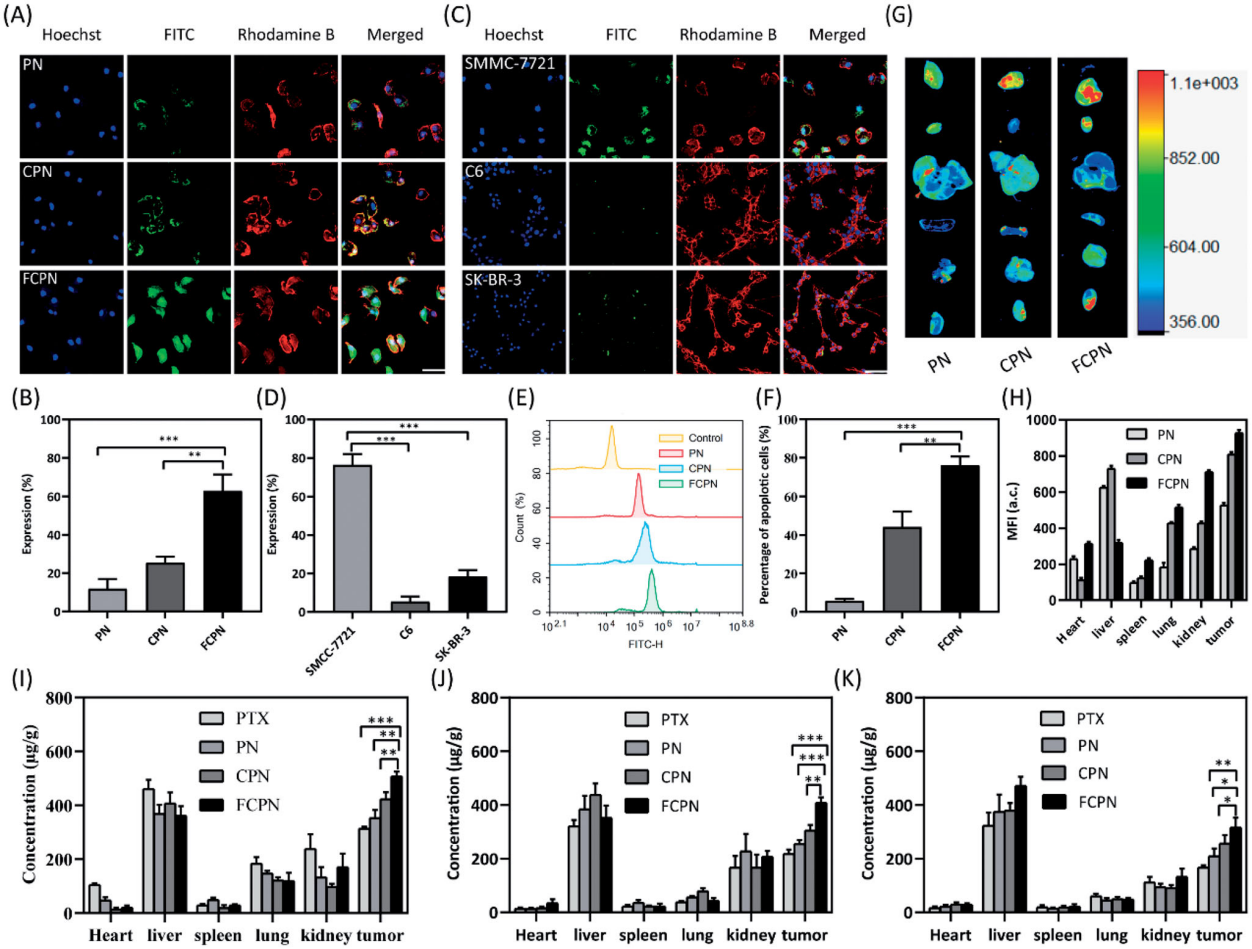
(A) CLSM cellular uptake images of FITC-labeled PN, CPN, and FCPN. Scale bars: 50 μm. (B) The fluorescence intensity of CLSM images in A. (C) CLSM cellular images of FCPN uptaken by SMMC-7721 cells, SK-BR-3 cells, and C6. Scale bars: 50 μm. (D) The fluorescence intensity of CLSM images in C. (E, F) Cellular uptake results by flow cytometry. (G) Tissue fluorescence imaging of FITC-labeled PN, CPN, and FCPN. (H) The fluorescence intensity in different organizations. (I–K) The drug biodistribution patterns of PTX, PN, CPN, and FCPN at 2 h, 10 h, and 24 h in tumor and main organs (heart, liver, spleen, lung, and kidney). All data represented the mean ± SD (*n* = 3) (*p<.05; **p<.01; ***p<.001).

Confocal laser scanning microscopy and flow cytometry were used to verify the targeting of FCPN at the cellular level. In the cellular uptake study, the CLSM images are shown in Figure 3(A,B). The nucleus and cytoskeleton were dyed with blue Hoechst 33342 and red rhodamine phalloidin, respectively. FITC-labeled PN, CPN, and FCPN produced green fluorescence. According to the fluorescence images, PN, CPN, and FCPN were all successfully taken up by SMMC-7721 cells, and compared with PN, the fluorescence intensities of the nanocrystals coated with the CM increased significantly. FCPN had the best cell uptake effect. As a control, the human breast cancer cell line SK-BR-3 and mouse glioma cell line C6 did not exhibit specific uptake of FCPN Figure 3(C,D). This indicated that after PN was coated with the SMMC-7721 CM, the targeting ability was improved due to the homologous binding ability of the CM with the same type. Furthermore, FA introduced as a targeted ligand further enhanced the active targeting of PN. The results of flow cytometry in Figure 3(E,F) were consistent with CLSM. This revealed that folate-functionalized membrane-coated nanocrystals exhibited excellent cellular uptake. Tissue fluorescence imaging and drug tissue distribution experiments further verified the targeting of FCPN at the animal level. After FITC-labeled PN, CPN and FCPN were injected into tumor-bearing mice for 3 h, and the fluorescence signals of the main organs are shown in Figure 3(G,H). The fluorescence intensities of the tumors in the FITC-labeled CPN group were higher than those of the FITC-labeled PN group, and the tumors of the FCPN group had the highest fluorescence intensities. Moreover, the ratio of fluorescence intensity between the tumor and liver also explained the good targeting of FCPN. This suggested the potential of folate-functionalized membranes as targeting materials. The biodistribution patterns of PTX, PN, CPN, and FCPN at 2 h, 10 h, and 24 h are shown in Figure 3(I–K). The PTX concentration in the tumor tissues of the PN group was higher than that of the PTX group. This was due to effective passive targeting of PN. The concentration of PTX in the tumor tissues of the CPN group and FCPN group was higher than that of the PN group at different time points because of the active targeting ability of the CM. Moreover, the effect of the FCPN group was better than that of the CPN group at different time points. The reason was that FA further enhanced the active targeting ability of the FCPN group. These results demonstrated that FCPN significantly increased the tumor accumulation of PTX, confirming that FA-CM had excellent tumor targeting and could increase PTX delivery to tumors. Meanwhile, the PTX concentration in the FCPN group decreased more slowly than that in the other groups, indicating that FCPN had a lower elimination rate, which could result in a longer systemic circulation time and allow it to play a longer therapeutic role. In addition, the liver was still the most significant drug metabolizing organ. The concentration of PTX was the highest in the liver compared with the other organs.

### 3.3. Apoptosis analysis

**Figure 4.**
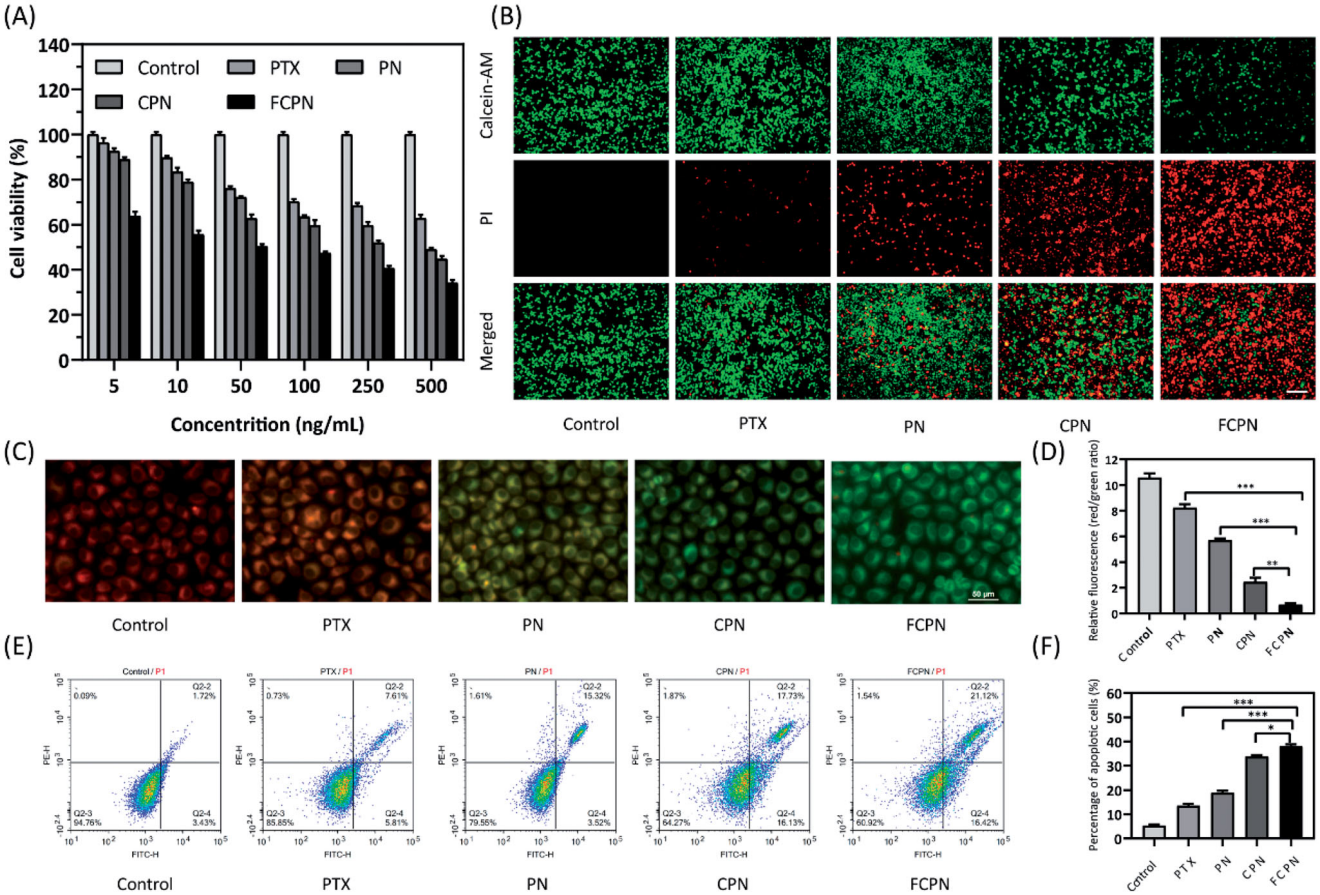
(A) The cell viability of SMMC-7721 cells after incubated with PTX, PN, CPN, and FCPN. (B) Fluorescence images of live and dead cells stained with Calcein-AM/PI after different treatments. Green fluorescence indicates live cells, red fluorescence indicates dead cells. Scale bars: 50 μm. (C) Fluorescence image of SMMC-7721 cells stained with JC-1 after different treatments. Scale bars: 50 μm. (D) The ratio changes of red fluorescence intensity to green fluorescence intensity in C represented the dissipation of MMP. (E, F) Flow cytometry analysis of apoptosis ratio and statistical analysis. All data represented the mean ± SD (*n* = 3) (*p<.05; **p<.01; ***p<.001).

**Figure 5.**
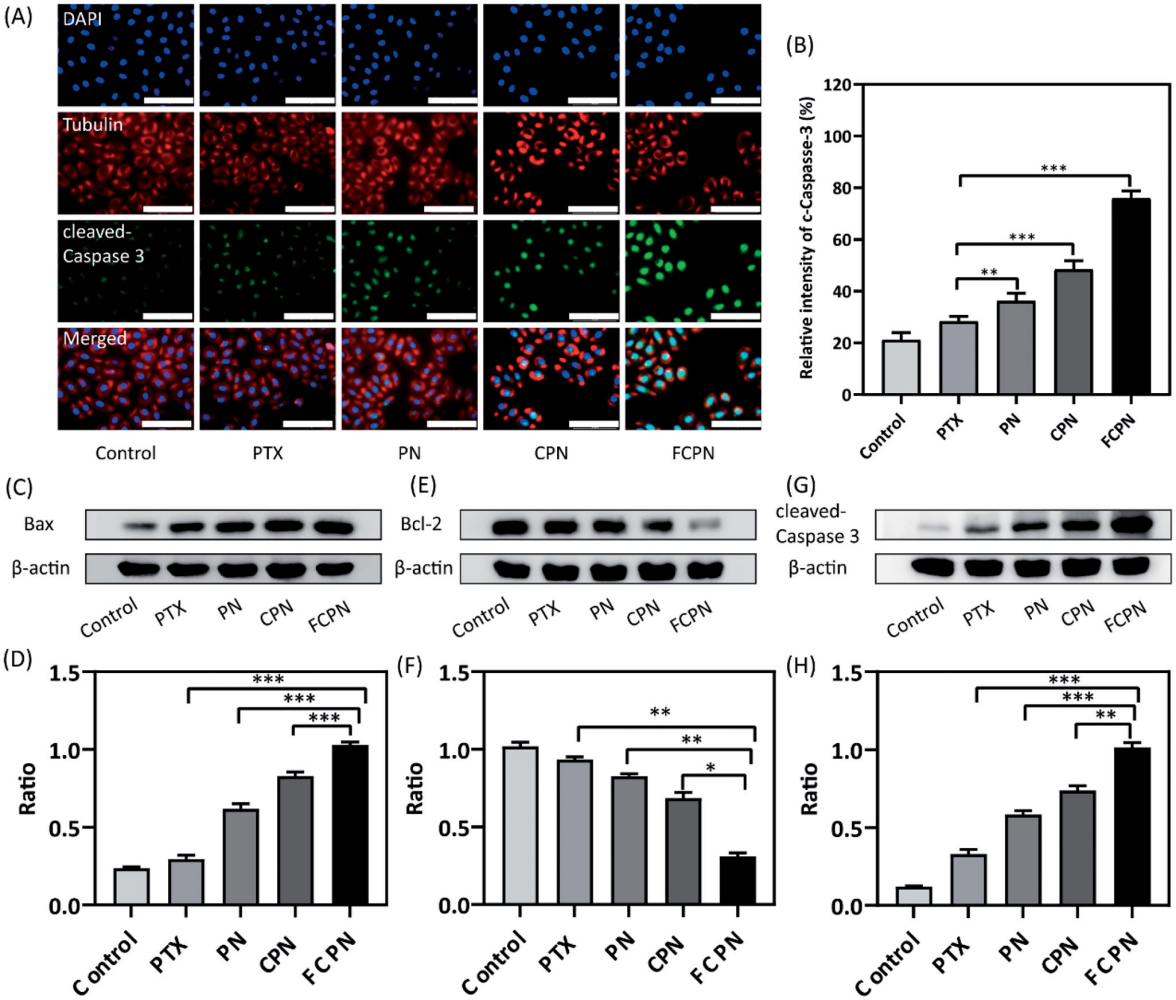
(A, B) Cleaved caspase-3 immunofluorescence images and intensity quantitative analysis of SMMC-7721 cells after different treatments. Cleaved caspase-3 apoptotic protein was stained green fluorescence. Cell nuclei were stained blue fluorescence with DAPI. cell cytoskeleton was stained red fluorescence with tubulin. Scale bars: 50 μm. (C, D) Bax protein expressions assayed by western blot after different treatments. (E, F) Bcl-2 protein expressions assayed by western blot after different treatments. (G, H) cleaved caspase-3 protein expressions assayed by western blot after different treatments. All data represented the mean ± SD (*n* = 3) (*p<.05; **p<.01; ***p<.001).

The toxicity of PTX, PN, CPN, and FCPN to SMMC-7721 liver cancer cells was evaluated by the MTT test. The cytotoxicity of different treatment groups is shown in Figure 4(A). When the drug concentration was 100 ng/mL, the cell viability of FCPN (48.89 ± 2.49%) was significantly reduced compared with that of PTX (77.64 ± 2.26%), PN (71.56 ± 2.44%), and CPN (62.4 ± 2.35%). With increasing PTX concentration, the cytotoxicity of different treatment groups gradually increased, and FCPN showed the greatest inhibitory effect. When the PTX concentration reached 500 ng/mL, the cell viability of FCPN was 37.81 ± 3.28%, while the cell viability of pure PTX, PN, and CPN was 64.63 ± 2.11%, 48.46 ± 1.80%, and 45.73 ± 3.00%, respectively. The IC50 values of PTX, PN, CPN, and FCPN were 952.54 ± 81.49 ng/mL, 437.86 ± 23.07 ng/mL, 275.46 ± 5.60 ng/mL, and 54.86 ± 9.17 ng/mL, respectively. MTT results showed that FCPN had strong tumor cytotoxicity. The fluorescence results confirmed the apoptosis-promoting effect of FCPN. In Figure 4(B), calcein-AM and PI were used to stain live and dead cells by exhibiting green fluorescence and red fluorescence, respectively. Fluorescent images showed that in the control group, all cells fluoresced green, indicating that there was no apoptosis. In contrast, the water solubility of PN was enhanced significantly, which promoted an increase in apoptotic cell number, and then the red fluorescence increased. After CM (CM or FA-CM) coating, CPN and FCPN acquired homologous targeting capability. The green fluorescence of the CPN and FCPN groups decreased significantly, while the red fluorescence increased significantly. The effect was particularly significant in the FCPN group. FCPN has a strong proapoptotic effect on tumor cells. JC-1 is an ideal fluorescent probe widely used to detect MMP to characterize mitochondrial function, which is closely related to cell apoptosis. When MMP is high, JC-1 gathers in the matrix of the mitochondria to form J-aggregates, which can produce red fluorescence. When NMP is low, JC-1 cannot form J-aggregates in the matrix of mitochondria. At this time, JC-1 is a monomer that produces green fluorescence. When the cell is apoptotic, MMP will decrease, thus producing green fluorescence. The decrease in the ratio of red fluorescence to green fluorescence represents the dissipation of MMP. According to Figure 4(C,D), the red/green fluorescence ratio of the FCPN group was the smallest compared with the other groups. This result further indicated that FCPN could significantly promote the apoptosis of SMMC-7721 cells, which was also consistent with the flow cytometry results. The apoptosis of SMMC-7721 cells treated with different pharmaceutical preparations for 48 h is shown in Figure 4(E,F). The apoptosis rate of cells treated with FCPN was 38.04 ± 0.81%. This was significantly higher than the CPN group of 33.81 ± 0.57%, the PN group of 18.93 ± 0.73%, and the PTX group of 13.52 ± 0.79%. This indicated that the efficacy of FCPN is definite at the cellular level *in vitro*. To more fully verify the proapoptotic effect, the immunofluorescence and western blot analyses of the expression of apoptotic proteins were performed. The cleaved caspase-3 green fluorescence intensity of the FCPN group in Figure 5(A,B) increased significantly compared with that of the CPN group, the PN group, and the PTX group. The western blot results in Figure 5(C–H) also showed that the expression of the apoptotic proteins Bcl-2 and cleaved caspase-3 in the FCPN group was the most striking, and the expression of Bax was the weakest. Bcl-2 family proteins (Bcl-2 and Bax) are two essential regulators of mitochondrial apoptosis and can regulate the opening of membrane channels and the flow of pro-apoptotic substances. Bax has a proapoptotic effect, and Bcl-2 can inhibit cancer cell apoptosis. Cleaved caspase-3 can destroy cell function and promote cell apoptosis. The combination of Bax/Bcl-2 can form an apoptotic dimer. When Bax/Bcl-2 increases, it will promote the release of apoptosis factors and cascade with Caspase protein to induce apoptosis (D'Orsi et al., 2017; Zhu et al., 2018). The Bax/Bcl-2 ratio of the FCPN group was still the largest, suggesting that it indeed had the best proapoptotic effect on cancer cells. These results clearly confirmed the potential of FCPN as an antitumor agent.

### 3.4. Antitumor effect and safety

**Figure 6.**
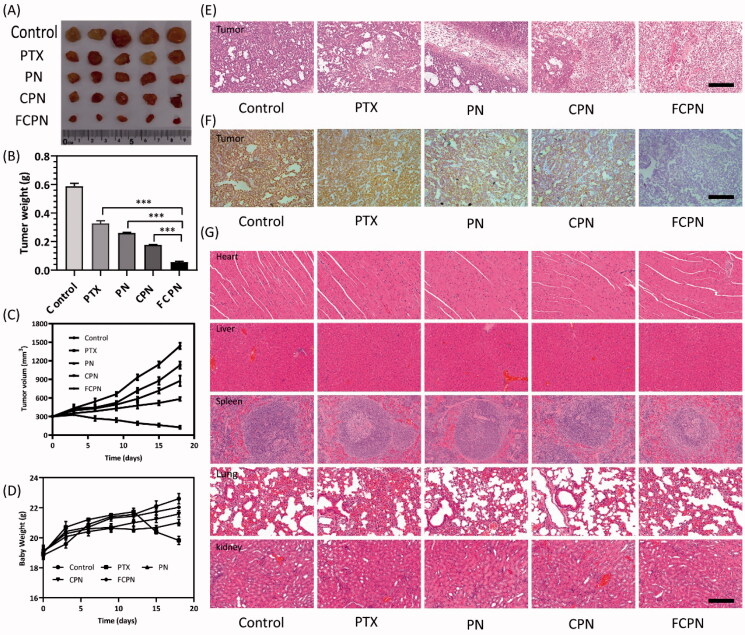
(A) Tumor images, (B) tumor weight, (C) tumor volume, and (D) body weight of different treatment groups after 21 days of treatment in different groups. (E) Tumor H&E staining in different treatment groups. Scale bars: 100 μm. (F) Tumor Ki67 immunohistochemical imaging in different treatment groups. Scale bars: 100 μm. (G) HE staining of the main organs in different treatment groups. Scale bars: 100 μm. All data represented the mean ± SD (*n* = 3) (***p<.001).

The antitumor effect and *in vivo* safety of FCPN are shown in Figure 6. Tumor-bearing nude mice were sacrificed at 21 d after administration, and the tumor tissues were removed and photographed, as shown in Figure 6(A). From the photos, we observed that the tumor volume of the FCPN group was significantly smaller than that of the other groups. The tumor weight of the FCPN group was 56.22 ± 5.42 mg, which was significantly lower than 176.74 ± 3.41 mg, 259.66 ± 5.56 mg, and 327.40 ± 17.79 mg of CPN, PN, and PTX groups in Figure 6(B). The FCPN group tumor volume was 126.51 ± 23.86 mm^3^, which was significantly reduced compared with the 584.30 ± 29.04 mm^3^, 876.42 ± 83.72 mm^3^, and 1129.21 ± 59.27 mm^3^ of the CPN, PN, and PTX groups in Figure 6(C). The tumor inhibition rates of the FCPN, CPN, PN, and PTX groups were 90.18 ± 0.88%, 70.02 ± 1.01%, 56.00 ± 1.53%, and 45.16 ± 3.39%, respectively. These results further proved that FCPN could significantly inhibit tumor growth. The weight change of the mice after 21 days of administration is shown in Figure 6(D). The weight of mice in the PTX group increased at the initial stage and then decreased gradually as time extended, indicating that it has obvious toxic side effects. The body weight of the preparation groups increased gradually, and the FCPN group still had the most significant effect. This suggested that functionalized membrane-coated nanocrystals significantly ameliorated drug side effects due to homologous targeting. The tumor H&E staining in Figure 6(E) confirmed that the FCPN group had significant necrosis compared with the CPN, PN, and PTX groups, which suggested that FCPN significantly promoted tumor cell apoptosis. Antigen Ki67 immunohistochemical staining in Figure 6(F) showed that the brown nuclei of proliferative cells in the FCPN group were significantly less abundant than those of the other groups. This further confirmed the prominent antitumor activity of FCPN *in vivo*. In Figure 6(G), histological tissue sections of the heart, liver, spleen, lungs, and kidneys showed that there were no significant differences between the groups, which confirmed that FCPN has better safety *in vivo*.

## 4. Conclusions

In this study, a bionic nanodrug system (FCPN) for targeted therapy of liver cancer was successfully prepared by CM coating technology. It had high drug loading and exhibited sustained release. Cell experiments showed that FCPN increased cell uptake, promoted cell apoptosis, and demonstrated that it had a good targeting effect on SMMC-7721 cells. *In vivo* animal experiments further proved that FCPN significantly improved bioavailability and increased drug accumulation in tumor tissues, which effectively inhibited tumor growth. This indicated that FA-CM was a promising active targeting material for the functionalization of particles.

## Supplementary Material

Supplemental Material

## Data Availability

The raw data required to reproduce these findings cannot be shared at this time as the data also form part of an ongoing study. The processed data (use in this manuscript) required to reproduce these findings can be shared at this time through personal request.
