# Supplementary material for: Folate-functionalized SMMC-7721 liver cancer cell membrane-cloaked paclitaxel nanocrystals for targeted chemotherapy of hepatoma
Source: Drug Deliv. 2021 Dec 28;29(1):31–42. doi: 10.1080/10717544.2021.2015481 (PMC8725828; doi:10.1080/10717544.2021.2015481)
Supplement: Supplemental Material [file IDRD_A_2015481_SM0912.docx]

**Supplement information**

**Folate-functionalized SMMC-7721 liver cancer cell membrane-cloaked paclitaxel nanocrystals for targeted chemotherapy of hepatoma**


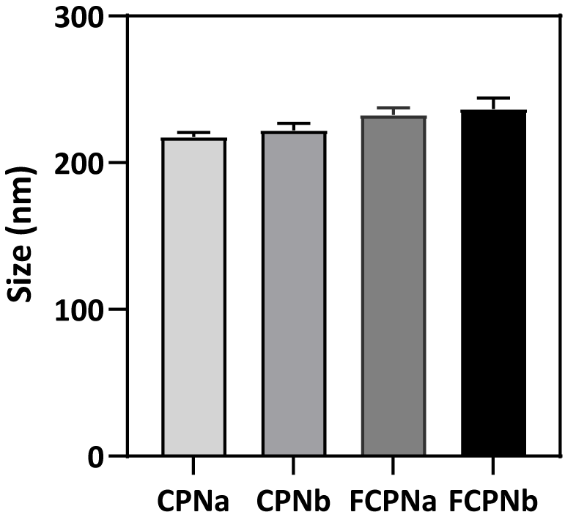


Fig S1. The particle size change of the samples before and after freeze drying. (CPNa and FCPNa represented the samples before freeze drying. CPNb and FCPNb represented the samples after freeze drying.)


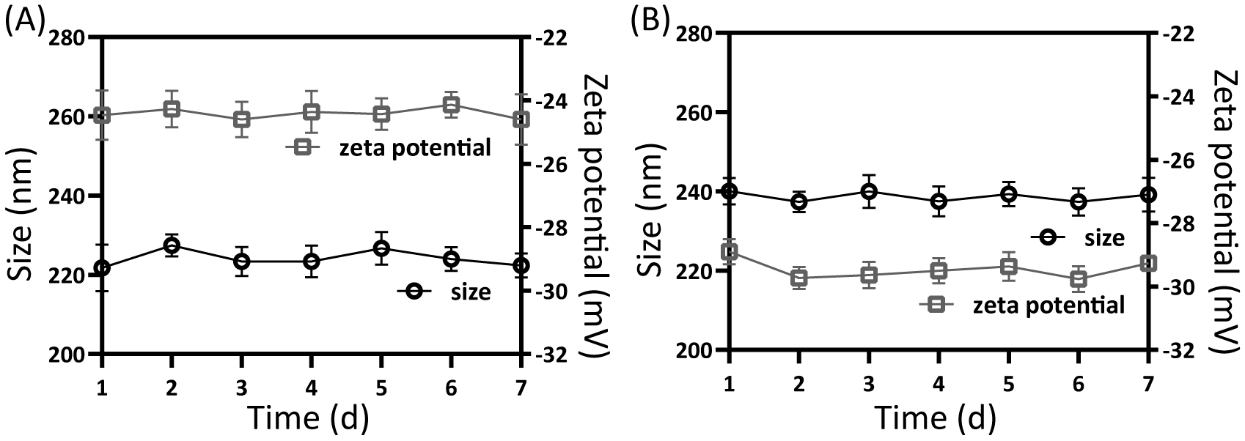


Fig S2. The 7-day stability of (A) CPN and (B) FPCN
